# The bmi-1 oncoprotein is differentially expressed in non-small cell lung cancer and correlates with INK4A-ARF locus expression

**DOI:** 10.1054/bjoc.2001.1791

**Published:** 2001-05

**Authors:** S Vonlanthen, J Heighway, H J Altermatt, M Gugger, A Kappeler, M M Borner, M van Lohuizen, D C Betticher

**Affiliations:** Department of Clinical Research,Institute of Pathology,Institute of Medical Oncology, University of Bern,Pathologie Länggasse, 3010 Bern, Switzerland; Gene Function Group, Roy Castle International Centre for Lung Cancer Research, Liverpool, UK; 6Division of Molecular Carcinogenesis, The Netherlands Cancer Institute, 1068 CX Amsterdam, The Netherlands

**Keywords:** bmi-1, polycomb group gene, cell cycle, p16, p14ARF, NSCLC

## Abstract

Genes of the polycomb group function by silencing homeotic selector genes that regulate embryogenesis. In mice, downregulation of one of the polycomb genes, bmi-1, leads to neurological alterations and severe proliferative defects in lymphoid cells, whilst bmi-1 overexpression, together with upregulation of myc-1, induces lymphoma. An oncogenic function has been further supported in primary fibroblast studies where bmi-1 overexpression induces immortalization due to repression of p16/p19ARF, and where together with H-ras, it readily transforms MEFs. It was the aim of this study to assess the expression of bmi-1 in resectable non-small cell lung cancer (NSCLC) in association with p16 and p14ARF (=human p19ARF). Tumours (48 resectable NSCLC (32 squamous, 9 adeno-, 2 large cell, 4 undifferentiated carcinomas and 1 carcinoid); stage I, 29, II, 7, III, 12; T1, 18, T2, 30; differentiation: G1 12, G2 19, G3 17) were studied by immunohistochemistry for protein expression and by comparative multiplex PCR for gene amplification analysis. In tumour-free, normal lung tissue from patients, weak – moderate bmi-1 staining was seen in some epithelial cells, lymphocytes, glandular cells and in fibroblasts, whereas blood, endothelial, chondrocytes, muscle cells and adipocytes did not exhibit any bmi-1 expression. In tumours, malignant cells were negative/weakly, moderately and strongly positive in 20, 22 and 6 cases, respectively. As assessed by multiplex PCR, bmi-1 gene amplification was not the reason for high-level bmi-1 expression. Tumours with moderate or strong bmi-1 expression were more likely to have low levels of p16 and p14ARF (*P* = 0.02). Similarly, tumours negative for both, p16 and p14ARF, exhibit moderate–strong bmi-1 staining. 58% of resectable NSCLC exhibit moderate–high levels of bmi-1 protein. The inverse correlation of bmi-1 and the INK4 locus proteins expression (p16/p14ARF) supports a possible role for bmi-1 misregulation in lung carcinogenesis. © 2001 Cancer Research Campaign www.bjcancer.com


*British Journal of Cancer* (2001) **84**(10), 1372–1376
© 2001 Cancer Research Campaign
doi: 10.1054/ bjoc.2001.1791, available online at http://www.idealibrary.com on IDEAL®

### The bmi-1 oncoprotein is differentially expressed in non-small cell lung cancer and correlates with INK4A-ARF locus expression

**S Vonlanthen[1], J Heighway[2], HJ Altermatt[3], M Gugger[4], A Kappeler[4], MM Borner[5], M van Lohuizen[6] and DC Betticher[5]**

[1]Department of Clinical Research, [4]Institute of Pathology, [5]Institute of Medical Oncology, University of Bern, [3]Pathologie Länggasse, 3010 Bern, Switzerland; [2]Gene Function Group, Roy Castle International Centre for Lung Cancer Research, Liverpool, UK; [6]Division of Molecular Carcinogenesis, The Netherlands Cancer Institute, 1068 CX Amsterdam, The Netherlands

**Summary** Genes of the polycomb group function by silencing homeotic selector genes that regulate embryogenesis. In mice, downregulation of one of the polycomb genes, bmi-1, leads to neurological alterations and severe proliferative defects in lymphoid cells, whilst bmi-1 overexpression, together with upregulation of myc-1, induces lymphoma. An oncogenic function has been further supported in primary fibroblast studies where bmi-1 overexpression induces immortalization due to repression of p16/p19ARF, and where together with H-ras, it readily transforms MEFs. It was the aim of this study to assess the expression of bmi-1 in resectable non-small cell lung cancer (NSCLC) in association with p16 and p14ARF (=human p19ARF). Tumours (48 resectable NSCLC (32 squamous, 9 adeno-, 2 large cell, 4 undifferentiated carcinomas and 1 carcinoid); stage I, 29, II, 7, III, 12; T1, 18, T2, 30; differentiation: G1 12, G2 19, G3 17) were studied by immunohistochemistry for protein expression and by comparative multiplex PCR for gene amplification analysis. In tumour-free, normal lung tissue from patients, weak – moderate bmi-1 staining was seen in some epithelial cells, lymphocytes, glandular cells and in fibroblasts, whereas blood, endothelial, chondrocytes, muscle cells and adipocytes did not exhibit any bmi-1 expression. In tumours, malignant cells were negative/weakly, moderately and strongly positive in 20, 22 and 6 cases, respectively. As assessed by multiplex PCR, bmi-1 gene amplification was not the reason for high-level bmi-1 expression. Tumours with moderate or strong bmi-1 expression were more likely to have low levels of p16 and p14ARF ($P = 0.02$). Similarly, tumours negative for both, p16 and p14ARF, exhibit moderate–strong bmi-1 staining. 58% of resectable NSCLC exhibit moderate–high levels of bmi-1 protein. The inverse correlation of bmi-1 and the INK4 locus proteins expression (p16/p14ARF) supports a possible role for bmi-1 misregulation in lung carcinogenesis. © 2001 Cancer Research Campaign http://www.bjcancer.com

**Keywords**: bmi-1; polycomb group gene; cell cycle; p16; p14ARF; NSCLC

The human proto-oncogene bmi-1 is a member of the mammalian Polycomb Group (Pc-G) family genes which are required during development to maintain stable repression of specific target sequences, such as homeo-box-cluster genes (Paro, 1995; Gould, 1997; van Lohuizen, 1998). bmi-1 was identified as one of the first Pc-G genes (Brunk et al, 1991; van Lohuizen et al, 1991b) by retroviral insertional mutagenesis when Eμ-myc transgenic mice were infected with Moloney murine leukaemia virus (bmi-1: *B*-cell specific *M*oloney murine leukaemia virus *i*nsertion site *1*) leading to a marked decrease in the latency period preceeding the development of pre-B-cell lymphomas in mice from approximately 150 to 50 days. Analysis of these tumours showed a variety of retroviral insertion sites with frequent integration near bmi-1, resulting in its overexpression (Haupt et al, 1991; van Lohuizen et al, 1991a). In vitro data has shown that bmi-1 overexpression in normal/primary human fibroblasts yields a proliferative advantage and delays the Hayflick limit in TIG3 cells (Jacobs et al, 1999; Voncken et al, 1999). Conversely, bmi-1$^{-/-}$mice suffer from neurological and severe proliferative defects in lymphoid cells (van der Lugt et al, 1994; Alkema et al, 1997b). Mouse embryonic fibroblasts deficient for bmi-1 expression show impaired progression in the S phase of the cell cycle and premature senescence.

It has been recently reported (Jacobs et al, 1999) that the INK4a-ARF tumour suppressor locus is a critical downstream target of bmi-1. Specifically, bmi-1 acts as a negative regulator of the INK4a-ARF locus, which encodes the two tumour suppressors p16INK4a and p14ARF (=human p19ARF). p16INK4a inhibits cell cycle progression by inhibiting cyclin D1-dependent kinases and thereby prevents the phosphorylation of the tumour suppressor Rb (Whyte, 1995) whereas p14ARF prevents the degradation and inactivation of the tumour suppressor p53 by binding to mdm-2 (Pomerantz et al, 1998; Zhang et al, 1998; Weber et al, 1999). Overexpression of bmi-1 allows fibroblast immortalization, downregulates expression of p16INK4a and p14ARF and, in combination with H-ras, leads to neoplastic transformation. Removal of the INK4a-ARF locus dramatically reduces the lymphoid and neurological defects seen in bmi-1$^{-/-}$mice, indicating that the INK4a-ARF locus is a critical in vivo target for bmi-1. Such a hypothesis is further supported by data correlating low levels of bmi-1 with markedly raised expression of p16INK4a and p14ARF in lymphocytes (Jacobs et al, 1999).

To test whether this regulatory mechanisms obtained in model systems might have relevance in primary human epithelial tumours, we initiated this study to assess the bmi-1 expression in a series of non-small cell lung cancer well documented for

*Received 29 September 2000*
*Accepted 28 February 2001*

*Correspondence to:* D Betticher p16INK4a, p14ARF, cyclin D1 and pRb expression. We show that bmi-1 is differentially expressed and that protein levels in the tumour cells are associated with the INK4a-ARF locus expression.

### PATIENTS, MATERIAL AND METHODS

### Patients' characteristics

Tumour samples were obtained from 48 patients (45 men, 3 women, median age of 65 years (45–79)) who had undergone resection at our hospital. They had received no therapy prior to surgery. 29 patients were in stage I, 7 in stage II and 12 in stage III: 32 were squamous cell carcinomas, 9 adenocarcinomas, 4 undifferentiated NSCLC, 2 large cell carcinomas and 1 carcinoid tumour. 18 of 48 patients are alive with a median follow-up of 20 months (range 1–64).

### Cell lines

All cell lines were cultured in a humidified $CO_2$ incubator at 37˚C in RPMI 1640 or DMEM (Sigma, St. Louis, MO) supplemented with 10% fetal calf serum, 100 μg ml$^{-1}$ streptomycin and 100 U ml$^{-1}$ penicillin.

### Paraffin embedding of cell lines

Cells were fixed in 4% formaldehyde and embedded in 4% agarose. The agarose button was processed to paraffin wax and immunostained as tissue sections.

### Immunohistochemistry

For bmi-1, 4 μm formalin-fixed, paraffin-embedded sections were dewaxed, rehydrated, and boiled (microwave) in EDTA buffer (pH 8.0). Sections were stained with the monoclonal anti-bmi-1F6 antibody (1: 200; (Alkema et al, 1997a)). As an internal control for staining intensity we used lymphocytes, which have been shown to express bmi-1 protein. Staining intensity was assessed as follows: negative-weakly (– – +), moderately (++) and strongly (+++) positive. Slides were assessed blinded, without knowledge of the expression of p16INK4a and p14ARF.

The staining for p16INK4a and p14ARF was performed as described previously (Betticher et al, 1996, 1997; Vonlanthen et al, 1998).

### Western blot analysis

Cells and tumour tissues were lysed in a Nonidet P40 lysis buffer (50 mM Tris-HCl pH 7.5, 150 mM NaCl, 0.1% SDS, 0.5% v/v NP-40 and protease inhibitors (complete™, Roche, Basel, Switzerland)). 100 μg of total protein was size-fractionated by SDS-Page and transferred to nitrocellulose (Schleicher and Schuell, Riehen, Switzerland). Equal loading was controlled by staining the membrane with Ponceau S (Sigma, Buchs, Switzerland). The membrane was probed with a monoclonal anti-bmi-1-F6 antibody (Alkema et al, 1997a), 1:1500 in TBS-T containing 1% dried milk or a polyclonal anti-p19ARF antibody (RB-045, Neomarkers, 1:200 in TBS-T containing 2% dried milk), which recognizes also the human homologue p14ARF. Detection was performed using an enhanced chemiluminescence system (ECL, Amersham, Zurich, Switzerland) according to the manufacturer's recommendations

### Comparative multiplex PCR

Tumours were screened for amplification of the bmi-1 gene by comparative multiplex PCR. Test primers (Bmi3f 5′cagatggcat-tatgcttgttgtac, Bmi3r 5′gtaagcaaggctcaacatagct) were located in the 3′untranslated region of bmi-1. Control primers (PR1 5′ggtttgtttctcactcatatagc, PR2 5′gtaggacctcaaggtgtagc) were located in genomic sequence flanking the progesterone receptor gene on chromosome 11q22–23. 50 μl PCR reactions contained four primers (Bmi3f, 0.5 μg; Bmi3r, 0.5 μg; PR1, 0.125 μg; PR2, 0.125 μg), 1× Taq reaction buffer (Roche), 250 μM dNTPs (Roche) and 1.25 units of Taq polymerase. Reactions were cycled 30 times in a PE Applied Biosystems GeneAmp PCR System 9700 machine at 58˚C for 1 min, 74˚C for 1 min and 94˚C for 1 min, with a final cycle of 58˚C for 1 min, 74˚C for 3 min. Products were visualized on 2.75% TBE agarose gels. The PCR reaction is competitive, with an amplification of either gene leading to a reduction in the PCR product intensity of the other. Reactions were balanced (by adjusting the initial primer concentration) to give approximately equal amounts of test and control products from a DNA target isolated from histologically normal lung tissue. The ratio of test to control bands derived from the tumour samples was assessed visually with reference to the control reactions.

### Statistical analyses

Associations of group membership with tumour characteristics were made with Fisher's exact tests for categorical features and the Mann–Whitney non-parametric test for continuous variables. Kaplan–Meier survival function estimates were used and simple comparisons between the two groups were made with the log-rank test.

### RESULTS AND DISCUSSION

### Bmi-1 expression in human cell lines

As a first step we studied the expression of bmi-1 in immunohistochemistry (IHC) experiments. Since the antibody has been shown to be specific in mice (Alkema et al, 1997a), the conditions for IHC for the bmi-1 monoclonal antibody were initially defined in human thymic and spleen tissues known to express high levels of bmi-1 (van Lohuizen et al, 1991a). Subsequently, the antibody was tested in a series of human cell lines by immunoblotting and IHC. In particular, 11 human cancer cell lines and 2 normal lung fibroblasts cell lines (IMR-90 and Wi-38) were prepared in the same manner as the tumours and examined for bmi-1 expression. Bmi-1 expression levels assessed by immunoblotting were in agreement with the IHC results in all cases. 4 cell lines expressed high levels of bmi-1 protein (leukaemia/lymphoma cells: Karpas 620, KG-1, Molt-4, and the breast MDA-MB 453 cell line). 5 further cell lines (K562 (leukaemia), MCF-7 (breast), Sa-OS-2 (bone), A549 and Calu-1 (lung)) exhibited moderate levels of bmi-1, and finally the 2 normal lung fibroblast cell lines (IMR-90 and Wi-38) as well as the HL-60 leukaemia line and Sk-ut-1B (uterus) were negative–very weakly positive for bmi-1 protein expression. In immunoblotting experiments, the antibody recognized up to 4 closely migrating protein bands of 40–44 kDa (Figure 1),

© 2001 Cancer Research Campaign

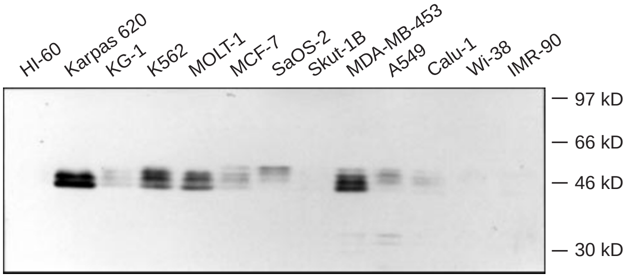

**Figure 1** Immunoblotting of bmi-1 in human cell lines

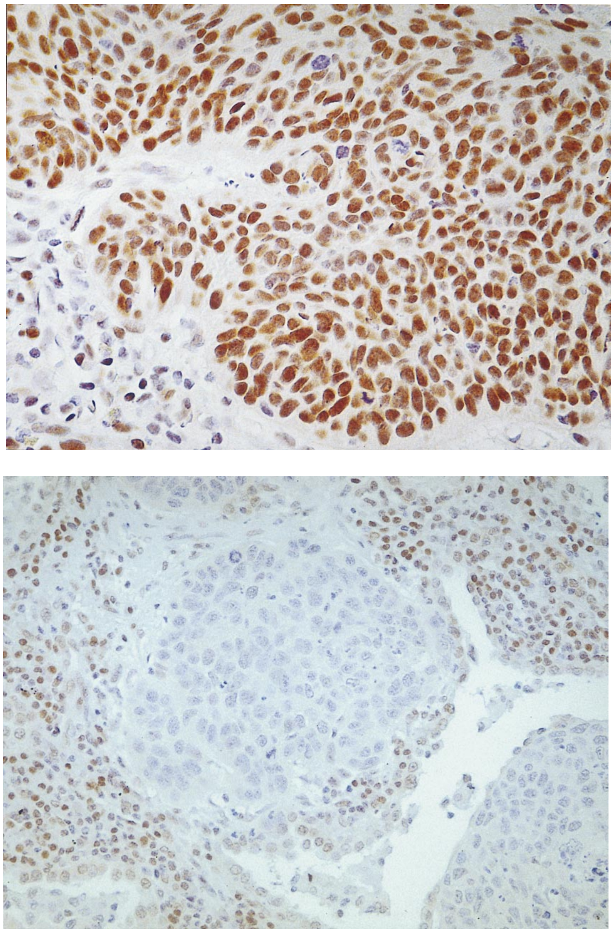

**Figure 2** Immunohistochemistry of bmi-1: tumour with positive (**A**) and negative (**B**) staining

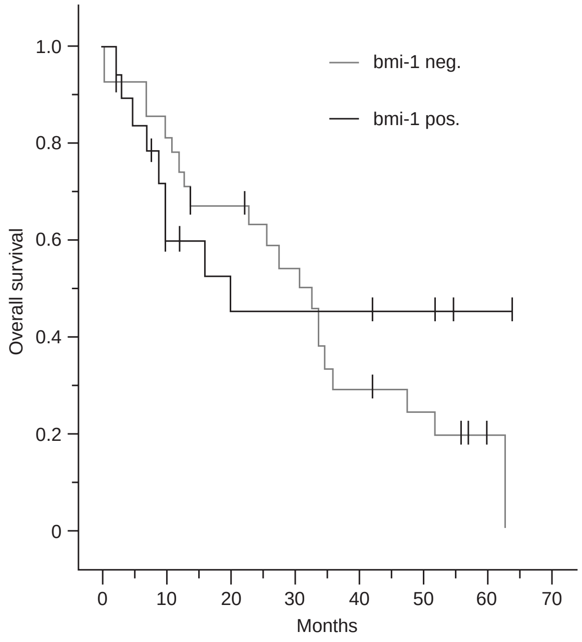

**Figure 3** Overall survival of patients with resectable NSCLC whose tumours expressed no–weak or moderate–strong levels of bmi-1

representing phosphorylated isoforms of the protein (Alkema et al, 1997a; Voncken et al, 1999). In conclusion, bmi-1 levels tend to be higher in cancer-derived human cell lines as compared to the 2 normal lung fibroblast lines. These results are in agreement with published data (Alkema et al, 1993; Lessard et al, 1998; Voncken et al, 1999).

### Bmi-1 expression in normal lungs and NSCLC

In normal lungs, stained by IHC, bmi-1 protein was weakly–moderately expressed in some epithelial cells, lymphocytes, glandular cells and in fibroblasts, whereas blood cells, endothelial, chondrocytes, muscle cells and adipocytes did not exhibit any bmi-1 expression. In tumours, malignant cells were negative–weakly, moderately or strongly positive in 20, 22 and 6 cases respectively (Figure 2 A, B). No association of bmi-1 protein expression with tumour criteria (proliferation rate, differentiation, size, histology classification) and patient's outcome were found (Figure 3), perhaps due to the low number of tumours examined. In conclusion, 58% of resectable NSCLC exhibit moderate to high levels of bmi-1. To our knowledge, this is the first description of an altered expression pattern of a Pc-G oncoprotein in human solid tumours.

In order to corroborate these IHC results in tumours, we performed immunoblotting analyses in several available tumour specimens. All tumours positive in IHC experiments exhibited a strong bmi-1 signal in immunoblots. Thus, it appears that both techniques, IHC and immunoblotting, can be used with this antibody for examining high-level bmi-1 expression in human malignant cells. However, we also observed a bmi-1 signal in immunoblots of some IHC-negative tumours, in particular if the lymphocytic infiltration was significant. Since we tested the antibody in cell lines (preparation identical as for tumours) and showed a strong association between immunoblotting and IHC, we assume that the bmi-1 bands obtained in IHC-negative tumours originate from the numerous contaminating non-malignant cells, in particular lymphocytes, expressing moderate levels of bmi-1.

### Associations of bmi-1 with INK4a-ARF locus expression

The present tumour series has been previously studied for p16INK4a (Betticher et al, 1997) and human p19ARF (=p14ARF) (Vonlanthen et al, 1998) expression. In order to corroborate the staining results we performed further immunoblotting in a subset of tumours that showed good correlation between immunoblotting and IHC (Figure 4). Regarding the recently described function of bmi-1, namely to regulate the INK4a-ARF locus, we looked for associations of bmi-1 and p16INK4a/p14ARF expression. In the present tumour series normal p16INK4a and p14ARF expression

 © 2001 Cancer Research Campaign

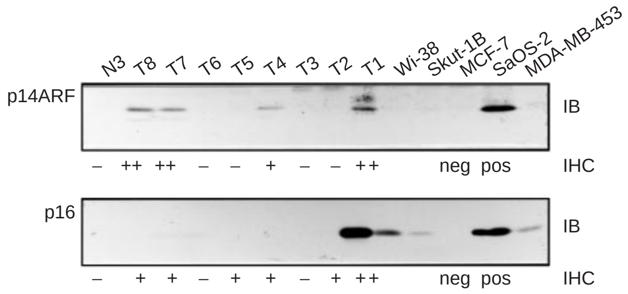

**Figure 4** Immunoblotting and results from IHC of p16INK4a and p14ARF in a subset of NSCLC and a series of cancer cell lines

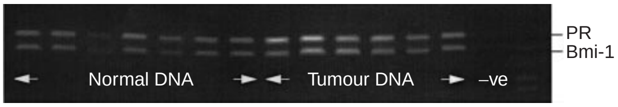

**Figure 5** PCR-multiplex of bmi-1 gene and progesterone receptor gene was found in 25/48 and 19/48, respectively (Vonlanthen et al, 1998). Some of the tumours exhibited normal p14ARF expression, whilst p16INKa was downregulated by strong methylation of the exon 1 alpha. Furthermore, a series of tumours (8/48) were negative for both p16INK4a and p14ARF expression. No methylation of exon 1 alpha nor mutations were found in the *CDKN2* (p16INK4a) gene. We were also unable to demonstrate homozygous loss at the *CDKN2* locus in any case (Betticher et al, 1997). Interestingly, these 8 tumours have strong–moderate bmi-1 expression. Out of a further 4 tumours, in which both proteins (p16INK4a and p14ARF) were downregulated and showed strong methylation of the exon 1 alpha, 3 also had bmi-1 expressed at high levels. Conversely, low levels of bmi-1 was associated with normal p16INK4a and p14ARF expression ($P$ = 0.02). These results support the hypothesis that increased expression of bmi-1 in primary human tumour cells leads to downregulation of the INK4a-ARF locus thereby impacting on both the p16INK4a-CDK4-cyclin D1-pRB and the p14ARF-mdm-2-p53 pathways.

### Bmi-1 gene amplification analysis in NSCLC

In order to investigate the mechanism underlying the overexpression of bmi-1 in resectable NSCLC, we examined whether the *bmi-1* gene on chromosome 10p13 was amplified. Previously, in 2 blastoid mantle cell lymphomas amplification of the *bmi-1* gene was found by Southern blot analysis (Bea et al, 1999). Comparative multiplex PCR was used to compare bmi-1 copy number to that of the progesterone receptor gene localized at 11q22–23. None of the 48 tumours showed an imbalance (favouring bmi-1) between test and control PCR products, suggesting that bmi-1 was not amplified in any of the lesions (Figure 3). It would therefore appear that an alternative mechanism(s) generates the bmi-1 overexpression. Candidates for such a mechanism include point mutation of control regions or coding sequence (leading to a stabilization of the protein), translocational upregulation, or alterations in transcription factor expression in the host cells. Further studies will be required to identify the particular cause of the overexpression of bmi-1 in lung carcinomas.

In conclusion, our results show that one of the polycomb group proteins, bmi-1, is differentially expressed in resectable NSCLC. The finding that this expression variance is associated with the expression of tumour suppressors encoded by the INK4a-ARF locus supports existing hypotheses developed in mice models and further suggests a role in lung cancer pathogenesis for the deregulation of bmi-1.

### ACKNOWLEDGEMENT

This work was supported by the Swiss Cancer League (KFS 177–9–1995, SKL 129–7–1995 and KFS 703–8–1998) and the Roy Castle Foundation, UK (JH).

### REFERENCES

Alkema MJ, Wiegant J, Raap AK, Berns A and van Lohuizen M (1993) Characterization and chromosomal localization of the human proto-oncogene Bmi-1. *Hum Mol Genet* **2**: 1597–1603

Alkema MJ, Bronk M, Verhoeven E, Otte A, van't Veer LJ, Berns A and van Lohuizen M (1997a) Identification of bmi-1-interacting proteins as constituents of a multimeric mammalian polycomb complex. *Genes Dev* **11**: 226–240

Alkema MJ, Jacobs H, van Lohuizen M and Berns A (1997b) Perturbation of B and T cell development and predisposition to lymphomagenesis in Emu Bmi1 transgenic mice require the Bmi1 RING finger. *Oncogene* **15**: 899–910

Bea S, Ribas M, Hernandez JM, Bosch F, Pinyol M, Hernandez L, Garcia JL, Flores T, Gonzalez M, Lopez-Guillermo A, Piris MA, Cardesa A, Montserrat E, Miro R and Campo E (1999) Increased number of chromosomal imbalances and high-level DNA amplifications in mantle cell lymphoma are associated with blastoid variants. *Blood* **93**: 4365–4374

Betticher DC, Heighway J, Haselton PS, Altermatt HJ, Ryder WD, Cerny T and Thatcher N (1996) Prognostic significance of CCND1 (cyclin D1) overexpression in primary resected non-small cell lung cancer. *Br J Cancer* **73**: 294–300

Betticher DC, White GRM, Vonlanthen S, Liu X, Kappeler A, Altermatt HJ and Heighway J (1997) G1 control gene status is frequently altered in resectable non-small cell lung cancer. *Int J Cancer* **74**: 556–562

Brunk BP, Martin EC and Adler PN (1991) Drosophila genes Posterior Sex Combs and Suppressor Two of Zeste encode proteins with homology to the murine bmi-1 oncogene. *Nature* **353**: 351–353

Gould A (1997) Functions of mammalian Polycomb group and trithorax group related genes. *Curr Opin Gen Develop* **7**: 488–494

Haupt Y, Alexander WS, Barri G, Klinken SP and Adams JM (1991) Novel zinc finger gene implicated as myc collaborator by retrovirally accelerated lymphomagenesis in Eμ-myc transgenic mice. *Cell* **65**: 753–763

Jacobs JJL, Kieboom K, Marino S, DePinho RA and van Lohuizen (1999) The oncogene and Polycomb-group gene bmi-1 regulates cell proliferation and senescence through the ink4a locus. *Nature* **397**: 164–168

Lessard J, Baban S and Sauvageau G (1998) Stage-specific expression of polycomb group genes in human bone marrow cells. *Blood* **91**: 1216–1224

Paro R (1995) Propagating memory of transcriptional states. *Trends Genet* **11**: 295–297

Pomerantz J, Schreiber-Agus N, Liegeois NJ, Silverman A, Alland L, Chin L, Potes J, Chen K, Orlow I, Lee HW et al (1998) The Ink4a tumor suppressor gene product, p19arf, interacts with MDM2 and neutralizes MDM2's inhibition of p53. *Cell* **92**: 713–723

van der Lugt NM, Domen J, Linders K, van Roon M, Robanus-Maandag E, Te Riele H, van der Valk M, Deschamps J, Sofroniew M, van Lohuizen et al (1994) Posterior transformation, neurological abnormalities, and severe hematopoietic defects in mice with a targeted deletion of the bmi-1 proto-oncogene. *Genes Dev* **8**: 757–769

van Lohuizen M (1998) Functional analysis of mouse Polycomb group genes. *Cell Mol Life Sci* **54**: 71–79

van Lohuizen M, Verbeeck S, Scheijen B, Wientjens E, van der Gulden H and Berns A (1991a) Identification of cooporating oncogenes in Eμ-myc transgenic mice by provirus tagging. *Cell* **65**: 737–752

van Lohuizen M, Frasch M, Wientjens E and Berns A (1991b) Sequence similarity between the mammalian bmi-1 proto-oncogene and the Drosophila regulatory genes Psc and Su(z)2. *Nature* **353**: 353–355

© 2001 Cancer Research Campaign 

Voncken JW, Schweizer D, Aagaard L, Sattler L, Jantsch MF and van Lohuizen M (1999) Chromatin-association of the Polycomb group protein bmi-1 is cell cycle-regulated and correlates with its phophorylation status. *J Cell Sci* **112**: 4627–4639

Vonlanthen S, Heighway J, Tschan MP, Borner MM, Altermatt HJ, Kappeler A, Tobler A, Fey MF, Thatcher N, Yarbrough WG and Betticher DC (1998) Expression of p16INK4a/p16alpha and p19ARF/p16beta is frequently altered in non-small cell lung cancer and correlates with p53 overexpression. *Oncogene* **17**: 2779–2785

Weber JD, Taylor LJ, Roussel MF, Sherr CJ and Bar-Sagi D (1999) Nucleolar arf sequesters Mdm2 and activates p53. *Nat Cell Biol* **1**: 20–26

Whyte P (1995) The retinoblastoma protein and its relatives. *Semin Cancer Biol* **6**: 83–90

Zhang Y, Xiong Y and Yarbrough WG (1998) ARF promotes MDM2 degradation and stabilizes p53. ARF-INK4a locus deletion impairs both the Rb and p53 tumor suppression pathways. *Cell* **92**: 725–734

*© 2001 Cancer Research Campaign*
